# YAP and TAZ regulate Schwann cell proliferation and differentiation during peripheral nerve regeneration

**DOI:** 10.1002/glia.23949

**Published:** 2020-12-18

**Authors:** Haley Jeanette, Leandro N. Marziali, Urja Bhatia, Abigail Hellman, Jacob Herron, Ashley M. Kopec, Maria Laura Feltri, Yannick Poitelon, Sophie Belin

**Affiliations:** ^1^ Department of Neuroscience and Experimental Therapeutics Albany Medical College Albany New York USA; ^2^ Department of Biochemistry Hunter James Kelly Research Institute, Jacobs School of Medicine and Biomedical Sciences, University at Buffalo Buffalo New York USA; ^3^ Department of Neurology Hunter James Kelly Research Institute, Jacobs School of Medicine and Biomedical Sciences, University at Buffalo Buffalo New York USA

**Keywords:** myelin, nerve injury, Schwann cell, Taz, Yap

## Abstract

YAP and TAZ are effectors of the Hippo pathway that controls multicellular development by integrating chemical and mechanical signals. Peripheral nervous system development depends on the Hippo pathway. We previously showed that loss of YAP and TAZ impairs the development of peripheral nerve as well as Schwann cell myelination. The role of the Hippo pathway in peripheral nerve regeneration has just started to be explored. After injury, Schwann cells adopt new identities to promote regeneration by converting to a repair‐promoting phenotype. While the reprogramming of Schwann cells to repair cells has been well characterized, the maintenance of such repair phenotype cannot be sustained for a very long period, which limits nerve repair in human. First, we show that short or long‐term myelin maintenance is not affected by defect in YAP and TAZ expression. Using crush nerve injury and conditional mutagenesis in mice, we also show that YAP and TAZ are regulators of repair Schwann cell proliferation and differentiation. We found that YAP and TAZ are required in repair Schwann cells for their redifferentiation into myelinating Schwann cell following crush injury. In this present study, we describe how the Hippo pathway and YAP and TAZ regulate remyelination over time during peripheral nerve regeneration.

## Main Points

- YAP and TAZ are required for Schwann cells dedifferentiation, proliferation, and remyelination after peripheral nerve crush injury.

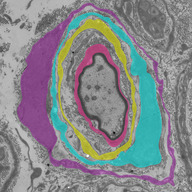

## 1 INTRODUCTION

In the vertebrate peripheral nervous system, Schwann cells (SCs) insulate axons and produce myelin, which allows rapid conduction of nerve impulses (reviewed in Salzer, 2015). In peripheral nerve injuries and diseases, SCs also possess the innate ability to dedifferentiate and transform into repair SCs to promote axonal regeneration and remyelination (reviewed in Jessen & Mirsky, 2019). Repair SCs are characterized by the loss of myelin proteins and the upregulation of multiple transcriptional mechanisms which enable the regeneration process. Notably, c‐JUN, ZEB2, mitogen‐activated protein kinase pathways, and chromatin modifications control and regulate the repair program in SCs (Arthur‐Farraj et al., 2012; He et al., 2018; Jacob, 2017; Parkinson et al., 2008; Quintes et al., 2016). Over the past years, numerous in vitro or in vivo studies in rodents have shown that axonal outgrowth can be accelerated using various approaches such as chemotactic cues, tissue engineering, scaffolds, electrical stimuli, mechanical stimuli, artificial nerve grafts; all helping neuronal guidance over relative short distances and short denervation duration (Dubey, Letourneau, & Tranquillo, 2001; Koliatsos, Clatterbuck, Winslow, Cayouette, & Price, 1993; Koppes et al., 2014; Rosen et al., 1990; Verdu et al., 2002). However, in humans, nerve regeneration remains challenging and often results in distal chronic denervation of SCs lasting for months or years (Fu & Gordon, 1995; Gordon, Tyreman, & Raji, 2011; Sulaiman, Midha, et al., 2002; Sulaiman & Gordon, 2000; Sulaiman, Voda, Gold, & Gordon, 2002). The failure of the PNS repair in human results from several factors, the slow axonal regrowth (1 mm/day), long nerve regrowth distance (up to 800 mm for Brachial plexus injuries), misrouting of axons with reinnervation errors, poor vascularization during regeneration, inhibitory cue in the extracellular matrix, failure to repopulate the injured site with SCs for the necessary extended period (reviewed in Hoke, 2006; Sulaiman & Gordon, 2009). In addition, remyelination by SCs that readopt a myelinating phenotype occurs at a slow rate and nerve function is compromised with a thinner myelin sheath (reviewed in Jessen & Mirsky, 2019). Thus, it is critical to better understand the signals regulating repair SCs to prolong their repair functions and improve axonal regeneration and remyelination.

Recent studies have demonstrated that SCs are sensitive to extracellular mechanical signals, which are critical for SC development and myelination (Belin, Zuloaga, & Poitelon, 2017; Fernando et al., 2016; Poitelon et al., 2016; Poitelon, Nunes, & Feltri, 2017; Urbanski et al., 2016). SCs translate external cues into biochemical signals that regulate gene expression by a process called mechanotransduction. Among the known mechanotransducers, YAP and TAZ, two paralogous transcriptional co‐activators of the Hippo pathway, have been the most extensively studied in SCs (reviewed in Belin et al., 2017). In the canonical Hippo pathway, MST1/2 kinases interact with SAV1 and phosphorylate LATS1/2, which phosphorylate YAP and TAZ on five (YAP) and four (TAZ) conserved serine residues (Feltri & Poitelon, 2018; Rausch & Hansen, 2020). Serine phosphorylations inhibit YAP and TAZ, resulting in their cytoplasmic retention and their ubiquitin‐mediated degradation (Meng, Moroishi, & Guan, 2016). Upon activation, YAP and TAZ are dephosphorylated and translocate into the nucleus where they regulate gene transcription. Like in other cell types, YAP and TAZ and the Hippo pathway activity can be regulated in SCs by transduction of cellular signals such as G‐protein and integrin signaling (Deng et al., 2017; Poitelon et al., 2016), SC polarization through CRB3 protein (Fernando et al., 2016), as well as mechanical cues such as stress of the F‐actin cytoskeleton, strain, cell density or stiffness of the extracellular environment (Poitelon et al., 2016). When YAP and TAZ are activated, they form multiple complexes with different DNA binding partners, but primarily with TEAD1–4. In SCs, TEAD1 has promyelinating functions and regulates essential myelin genes in cooperation with EGR2 and SOX10 (Deng et al., 2017; Grove et al., 2017; Lopez‐Anido et al., 2016; Poitelon et al., 2016). In contrast to TEAD1, TEAD4 was shown to be a repressor of myelination in SCs (He et al., 2018).

YAP and TAZ are often referred together as YAP/TAZ based on their sequence homology and structural similarities. However, these proteins can play different roles as illustrated by individual constitutive mouse knockout lines (Makita et al., 2008; Morin‐Kensicki et al., 2006). TAZ and YAP protein levels are respectively increased in *Yap* or *Taz* single‐knockout SCs during development, suggesting compensatory effects (Deng et al., 2017; Poitelon et al., 2016). Ablation of both YAP and TAZ decreases SC proliferation and limits SC differentiation into mature SCs (Deng et al., 2017; Grove et al., 2017; Poitelon et al., 2016). However, while TAZ protein levels increase during active myelination and decrease in adulthood, YAP protein levels are stable from birth to adulthood (Poitelon et al., 2016). In addition, while one allele of *Taz* is sufficient to compensate for *Yap* deficiency, one allele of *Yap* does not prevent severe peripheral nerve development defects caused by the absence of *Taz* in SCs. Thus, TAZ plays a more important role than YAP in SCs during development and active myelination (Deng et al., 2017; Poitelon et al., 2016).

After peripheral nerve injury, YAP protein expression is not affected and analysis of *Yap* single‐knockout SCs suggests that YAP alone is not required for peripheral nerve repair and remyelination (Mindos et al., 2017). However, TAZ protein expression is upregulated similarly to c‐JUN (Mindos et al., 2017) and analysis of *Taz* single‐knockout in SCs show that absence of TAZ limits peripheral nerve remyelination (Grove, Lee, Zhao, & Son, 2020). Therefore, TAZ also plays a more important role than YAP in SC remyelination. SCs lacking both YAP and TAZ (*Yap* and *Taz* double‐knockout) wrap around regenerated axons normally but fail to differentiate into myelinating SCs to remyelinate axons (Grove et al., 2020). Surprisingly, absence of YAP and TAZ was shown to have no effect on the proliferation of repair SC and on the expression level of the key SC dedifferentiation marker c‐JUN (Grove et al., 2020). Further, the role of YAP and TAZ in myelin maintenance is controversial due to conflicting reports, describing YAP and TAZ as being either essential or non‐essential for myelin maintenance when ablated in adult nerves (Deng et al., 2017; Grove et al., 2017). Those conflicting results issued from a YAP/TAZ model with a pre‐existing severe phenotype (Deng et al., 2017; Grove et al., 2017) motivated us to select a new model with a normal lifespan to clarify both short‐term and long‐term YAP and TAZ functions in myelin maintenance and nerve repair.

In this study, we used an inducible composite mouse model to selectively inactivate YAP and TAZ in SCs; *Yap* ^cHet^; *Taz* ^cKO^; *Plp1*‐Cre^ER^. This viable compound model helped us to clarify both short‐term and long‐term functions of YAP and TAZ in myelin maintenance and remyelination. Supported by molecular, morphological, nerve conduction electrophysiology and behavior results, we showed that while YAP and TAZ are dispensable for myelin maintenance, YAP and TAZ are major regulators of the SC injury early response. Loss of YAP and TAZ in SCs strikingly compromised remyelination and nerve functional repair. Notably, we showed that YAP and TAZ specify SC differentiation into myelinating SCs during nerve repair. Importantly, the long‐term effect of YAP and TAZ loss of function on remyelination was compensated over time.

## 2 MATERIALS AND METHODS

### 2.1 Animal model

All experiments involving animals followed experimental protocols approved by the Albany Medical College Institutional Animal Care and Use Committee. *Yap* fl/+; *Taz* fl/fl; *Plp1*‐Cre^ER^ and *Yap* fl/+; *Taz* fl/fl; *Sox10*‐CreER mice in a C57BL/6J background were generated. Tamoxifen was injected into adult *Yap* fl/+; *Taz* fl/fl; *Plp1*‐CreER and *Yap* fl/+; *Taz* fl/fl; *Sox10*‐Cre^ER^ mice. A 20 mg/ml solution of tamoxifen was prepared in corn oil. This solution was injected intraperitoneally at a concentration of 100 mg/kg body weight. Five injections were performed, every 12 hr. The resulting mutant mice were compared with their littermates injected with corn oil only. Genotyping of mutant mice was performed by PCR on tail genomic DNA (Poitelon et al., 2016). Animals were housed in cages of 5 in 12/12‐hr light/dark cycles. No animals were excluded from the study. Equal numbers of males and females were included in the study. Mutant and control littermates from both sexes were sacrificed at the indicated ages, and sciatic nerves were dissected.

### 2.2 Nerve crush

Nerve crush was performed on adult mice using aseptic technique under a laminar flow hood. Mice were anesthetized with isofluorane and eyes were lubricated. While the mouse laid flat on its dorsal side, the region of intended crush was shaved and cleaned with iodine and ethanol. On one leg, a small incision was made in the skin and muscle above the approximate position of sciatic nerve. Once nerve was exposed, any membranous ties to the muscle were severed using small scissors. A pair of hemostatic forceps were dipped in liquid N2 for 2 s and dipped in activated carbon after, then nerves were tightly compressed with chilled clamps for 60 s. Nerve was crushed, but not severed. Position of crush was approximately the same for every cohort. The other leg was unoperated. Injured skin was closed with a staple. Animals were monitored daily after surgery and analgesics were administered, as necessary. Then, 5, 7, 20, or 60 days postinjury, the crushed and lateral control nerves were sampled (Catignas et al., 2020).

### 2.3 Behavior analysis

For von Frey filament (VFF) analysis, animals were analyzed both at 20 and 60 days after injury, as described (Prabhala et al., 2018). The VFF test was used to assess mechanical threshold using the Dixon up‐down method (Dixon, 1991). Each mouse began receiving mechanical stimulation with the smallest VFF (1.65 mm, or 0.008 g). The 50% mechanical threshold was determined through Chaplan's formula (Chaplan, Bach, Pogrel, Chung, & Yaksh, 1994). For grip strength analyses, animals were analyzed 20 and 60 days after injury, as previously described (Poitelon et al., 2012). Forelimb and hind limb were tested lowering the mouse over the grid, keeping the torso parallel with the grid and allowing both forepaws and hind paws to attach to the grid before to start the measurement. Then, the mouse was gently pulled back by its tail following the axle of the sensor and the maximal grip strength value of each mouse was recorded. This measure was performed five times on each animal and the mean value was calculated. The results were normalized to the weight of the animal. For the rotarod analysis, animals were analyzed 60 days after injury as previously described (Della‐Flora Nunes et al., 2017). Control and mutant littermates were tested in two sessions of three trials each per day (6 hr rest between the two daily sessions) for three consecutive days.

### 2.4 Electrophysiological analyses

Animals were analyzed 20 and 60 days after injury as described previously (Poitelon et al., 2015; Poitelon et al., 2018). Mice were anesthetized with tribromoethanol, 0.4 mg g^−1^ of body weight, and placed under a heating lamp to avoid hypothermia. Motor conduction velocity and amplitude of sciatic nerve were obtained with subdermal steel monopolar needle electrodes: a pair of stimulating electrodes was inserted subcutaneously near the nerve at the ankle, then at the sciatic notch, and finally at the paraspinal region at the level of the iliac crest to obtain three distinct sites of stimulation, proximal and distal, along the nerve. Compound motor action potentials were recorded with an active electrode inserted in muscles in the middle of the paw and a reference needle in the skin between the first and second digits. Electrophysiological studies comprising motor and sensory nerve conduction studies were conducted using a VikingQuest electromyography device.

### 2.5 Morphological analysis

Mutant and control littermates were euthanized at the indicated ages, and sciatic nerves were dissected. Nerves were fixed in 2% buffered glutaraldehyde and postfixed in 1% osmium tetroxide. After alcohol dehydration, the samples were embedded in Epon. Transverse sections (0.5–1 nm thick) were stained with toluidine blue and examined by light microscopy. Ultrathin sections were stained with uranile acetate and lead citrate and examined by electron microscopy. For all morphological assessments, at least three animals per genotype were analyzed. For g ratio analysis of sciatic nerves (axon diameter/fiber diameter), semithin section images were acquired with a ×100 objective. G ratios were determined for at least 100 fibers chosen randomly per animal. Axon and fiber diameters, the number of myelinated fibers, and the number of onion bulbs were quantified on semithin sections using the ImageJ software (imagej.nih.gov/ij). Quantification axons in Remak bundles were quantified on ultrathin sections. Data were analyzed using GraphPad Prism 6.01. Images were blindly evaluated during analysis. Pseudocoloring was performed with Adobe Animate.

### 2.6 Western blotting

Contralateral and crush sciatic nerves were dissected, and the segment distal to the crush site was use for further analysis. Sciatic nerves were then frozen in liquid nitrogen, pulverized and resuspended in lysis buffer (150 mm NaCl, 25 mm HEPES, 0.3% CHAPS, pH 7.4, 1 mm Na3VO4, 1 mm NaF and 1:100 Protease Inhibitor Cocktail [Roche Diagnostic, Florham Park, NJ]) (Belin, Herron, et al., 2019; Belin, Ornaghi, et al., 2019). Protein lysates were centrifuged at 15,000 g for 30 min at 4°C. Supernatant protein concentrations were determined by bicinchoninic acid assay protein assay (Thermo Scientific, Waltham, MA) according to the manufacturer's instructions. Equal amounts of homogenates were diluted 3:1 in 4× Laemmli (250 mm Tris–HCl, pH 6.8, 8% sodium dodecyl sulfate (SDS), 8% β‐mercaptoethanol, 40% glycerol, 0.02% bromophenol blue), denatured 5 min at 100°C, resolved on SDS‐polyacrylamide gel and electroblotted onto PVDF membrane. Blots were then blocked with 5% bovine serum albumin in 1× phosphate‐buffered saline (PBS), 0.05% Tween‐20 and incubated overnight with the following appropriate antibodies: anti GAP43 1/2,000 (Abcam, ab16053), anti TAZ 1/1,000 (ProteinTech, 23306‐1‐AP), anti YAP 1/200 (Santa Cruz, sc‐101199), anti‐peripheral myelin protein (PMP2) 1/200 (Santa Cruz, sc‐374058), anti c‐JUN 1/1,000 (Cell Signaling, 60A8), anti P0 1/5,000 (Aves Labs, PZO), anti‐MBP 1/1,000 (Covance, SMI99), anti‐GAPDH 1/2,000 (Sigma, G9545), and anti‐Calnexin 1/3,000 (Sigma, C4731). Membranes were then rinsed in 1× PBS and incubated for 1 hr with secondary antibodies. Blots were developed using ECL or ECL plus (GE Healthcare, Chicago, IL). Western blots were quantified using ImageJ software (http://imagej.nih.gov/ij).

### 2.7 Immunohistochemistry

Contralateral and crush sciatic nerves were dissected, and the segment distal to the crush site was use for further analysis. Immunostaining of sciatic nerves: OCT‐embedded sciatic nerves were sliced into 10 μm‐thick sections with Cryostat and stored in −80°C. Sections were thawed for 5 min (Catignas et al., 2020). Nerve sections were permeabilized with −20°C 100% methanol, washed in PBS, then incubated in blocking solution for 1 hr RT. Nerves were incubated overnight at 4°C with primary Abs diluted in blocking solution. The following primary Abs were used: anti‐mouse pan macrophages F4/80 1/400 (Cedarlane CL89170AP[‐S]), anti pH 3‐488 conjugated 1/50 (Sigma, 06‐570‐AF488), anti Ki67 1/1,000 (Abcam, ab15580), anti c‐JUN total 1/2,000 (Cell Signaling, 60A8), anti‐EGR2 1/200 was gifted by Dr Meijer of the Centre for Neuroregeneration, Edinburgh. Nerves were washed, incubated with appropriate secondary Ab diluted in blocking solution for 1 hr at room temperature, washed, stained with DAPI [1/10,000] (Sigma‐Aldrich D9542) for 5 min room temperature, washed, mounted with Vectashield, then sealed. Images were acquired at ×20 with a Zeiss epifluorescent microscope. The total number of cells was counted along with the number of positive cells in each corresponding stain. Analysis was done using ImageJ Software (http://imagej.nih.gov/ij).

### 2.8 RNA preparation and quantitative RT‐PCR

Contralateral and crush sciatic nerves were dissected, and the segment distal to the crush site was use for further analysis. Sciatic nerves were dissected, stripped of epineurium, frozen in liquid nitrogen, pulverized and processed as described (Belin et al., 2019 b). Total RNA was prepared from sciatic nerve or SCs with TRIzol (Roche Diagnostic). One microgram of RNA was reverse transcribed using Superscript III (Invitrogen, Carlsbad, CA). For each reaction, 5 μM of oligo(dT)20 and 5 ng/μl random hexamers were used. Quantitative PCR was performed using the 20 ng of cDNA combined with 1× FastStart Universal Probe Master (Roche Diagnostic). Data were analyzed using the threshold cycle (Ct) and 2(−ΔΔCt) method. *Actb* was used as endogenous gene of reference and 18S was used as to validate the stable expression of *Actb*. The primers and probe used are the following: 18S (F: ctcaacacgggaaacctcac, R: cgctccaccaactaagaacg, Probe #77), mouse *Actb* (F: aaggccaaccgtgaaaagat, R: gtggtacgaccagaggcatac, Probe #56), mouse *Wwtr1* (Taz) (F: gcaacatggacgagatggat, R: gaaggcagtccaggaaatca, Probe #3).

### 2.9 Statistical analyses

Experiments were not randomized, but data collection and analysis were performed blind to the conditions of the experiments. Data are presented as mean ± *SEM* or *SD*. No statistical methods were used to predetermine sample sizes, but our sample sizes are similar to those generally employed in the field. Two‐tailed Student's *t* test was used for statistical analysis of the differences between multiple groups. Values of *p* ≤ .05 were considered to represent a significant difference.

## 3 RESULTS

### 3.1 YAP and TAZ are not required for myelin maintenance in sciatic nerve

Others and we showed that SCs lacking both YAP and TAZ are unable to proliferate properly and fail to myelinate developing peripheral nerves (Deng et al., 2017; Grove et al., 2017; Poitelon et al., 2016). Grove et al. recently reported that YAP and TAZ are also essential for functional regeneration of peripheral nerves using inducible *Yap* and *Taz* double knockout in SC; *Yap* fl/fl; *Taz* fl/fl; *Plp1*‐cre^ER^ (Grove et al., 2020). However, animals ablated for both *Yap* and *Taz* in adult myelinating SCs and oligodendrocytes exhibit severe weight loss, tremors, ataxia, and mortality within 2 weeks (Figure S1) (Deng et al., 2017; Grove et al., 2017), which prevents the assessment of the long‐term effect of YAP and TAZ loss required to make conclusions on their role in myelin maintenance and nerve regeneration after injury.

**FIGURE 1:**
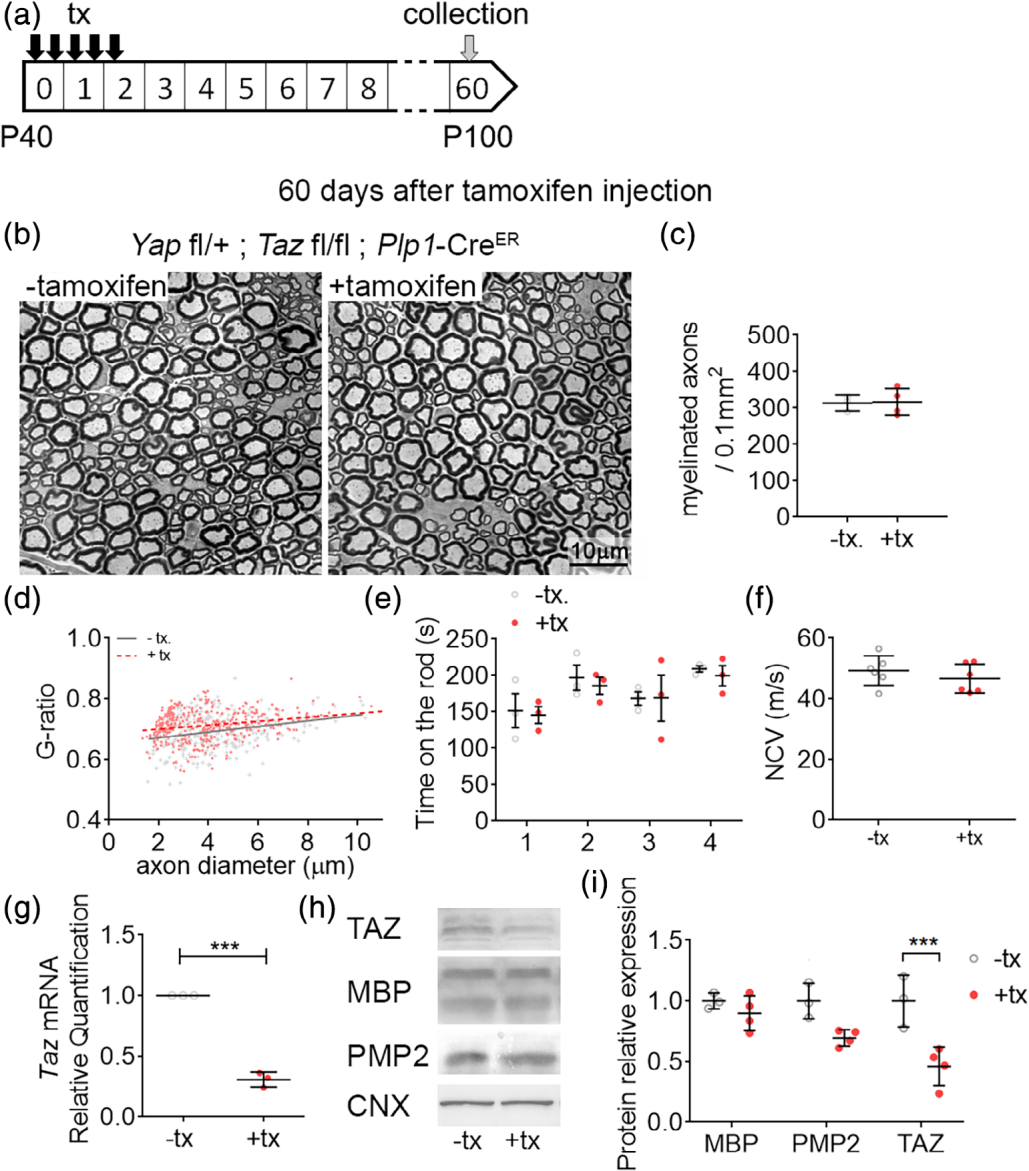
YAP/TAZ are dispensable for myelin maintenance. (a) Schematic showing experimental procedures analyzing *Yap* ^cHet^; *Taz* ^cKO^ intact sciatic nerves, 60 days after oil (−tx) or tamoxifen (+tx) injections. (b) Semithin sections of *Yap* ^cHet^; *Taz* ^cKO^ sciatic nerves 60 days after oil (−tx) or tamoxifen (+tx) injections. Bars, 10 μm. (c) Counts of numbers of myelinated fibers in *Yap* ^cHet^; *Taz* ^cKO^ sciatic nerves 60 days after oil (−tx) or tamoxifen (+tx) injections. (d) G ratio versus axon diameter scatter plot graphs from myelinated fibers in *Yap* ^cHet^; *Taz* ^cKO^ sciatic nerves 60 days after oil (−tx) or tamoxifen (+tx) injections. (e) Rotarod analysis of motor function in *Yap* ^cHet^; *Taz* ^cKO^ 60 days after oil (−tx) or tamoxifen (+tx) injections. (f) Measurements of nerve conduction velocity (NCV) from *Yap* ^cHet^; *Taz* ^cKO^ sciatic nerves 60 days after oil (−tx) or tamoxifen (+tx) injections. (g) RTq‐PCR of TAZ, MBP and PMP2 in *Yap* ^cHet^; *Taz* ^cKO^ sciatic nerves 3 days after oil (−tx) or tamoxifen (+tx) injections. TAZ mRNA levels are lower in *Yap* ^cHet^; *Taz* ^cKO^ (+tx) compared to *Yap* ^cHet^; *Taz* ^cKO^ (−tx). (h‐i) Western blot of TAZ, MBP and PMP2 in sciatic nerves 60 days after oil (−tx) or tamoxifen (+tx) injections. TAZ protein levels are lower in *Yap* ^cHet^; *Taz* ^cKO^ (+tx) compared to *Yap* ^cHet^; *Taz* ^cKO^ (−tx). Calnexin (CNX) is used a loading control. n ≥ 3 mice for each genotype and time point. Data are presented as means ± *SEM*. Two‐sided Student's *t* test: ***, *p* ≤ .001 [Color figure can be viewed at wileyonlinelibrary.com]

To clarify the role of YAP and TAZ in myelin maintenance in adult nerves, we generated compound models *Yap* fl/+; *Taz* fl/fl; *Plp1*‐cre^ER^ and *Yap* fl/+; *Taz* fl/fl; *Sox10*‐cre^ER^. Both *Plp1*‐cre^ER^ and *Sox10*‐cre^ER^ expression is conditional following intraperitoneal injections of tamoxifen and allows for the controlled ablation of YAP and TAZ in adult myelinated glial cells (both oligodendrocytes and SCs). These two mouse models are viable past 14 days posttamoxifen injection, allowing us to study the long‐term effect of YAP and TAZ loss of function on myelin maintenance. *Yap* fl/+; *Taz* fl/fl; *Plp1*‐cre^ER^; or *Sox10*‐cre^ER^ animals were injected at postnatal Day 30 with tamoxifen. *Taz* mRNA and TAZ protein levels were reduced in *Yap* fl/+, *Taz* fl/fl, *Plp1*‐cre^ER^ animals 3 days after the first tamoxifen injection (Figure 1g,h). The number of myelinated axons and myelin protein levels, such as myelin protein zero (P0) and PMP2, were not affected in animals ablated for YAP and TAZ in adult SCs 60 days after the first injection (Figures 1 b–d,h,i and S2). We also did not observe any effect on the motor behavior or nerve function of these animals (Figure 1e,f). These results demonstrate that the inducible compound ablation of YAP and TAZ in adult myelinating SCs does not affect myelin maintenance in the peripheral nervous system, at least up to 60 days after YAP and TAZ recombination. These results also reveal that *Yap* fl/+; *Taz* fl/fl; *Plp1*‐cre^ER^ animals (*Yap* ^cHet^; *Taz* ^cKO^) is a suitable model to study peripheral nerve regeneration and remyelination, as they do not present a preexisting phenotype before injury that could interfere with assessing YAP and TAZ function in nerve repair. *Yap* ^cHet^; *Taz* ^cKO^ animal are used in the rest of this study.

### 3.2 Loss of YAP and TAZ causes severe regeneration and functional defects after nerve injury

**FIGURE 2:**
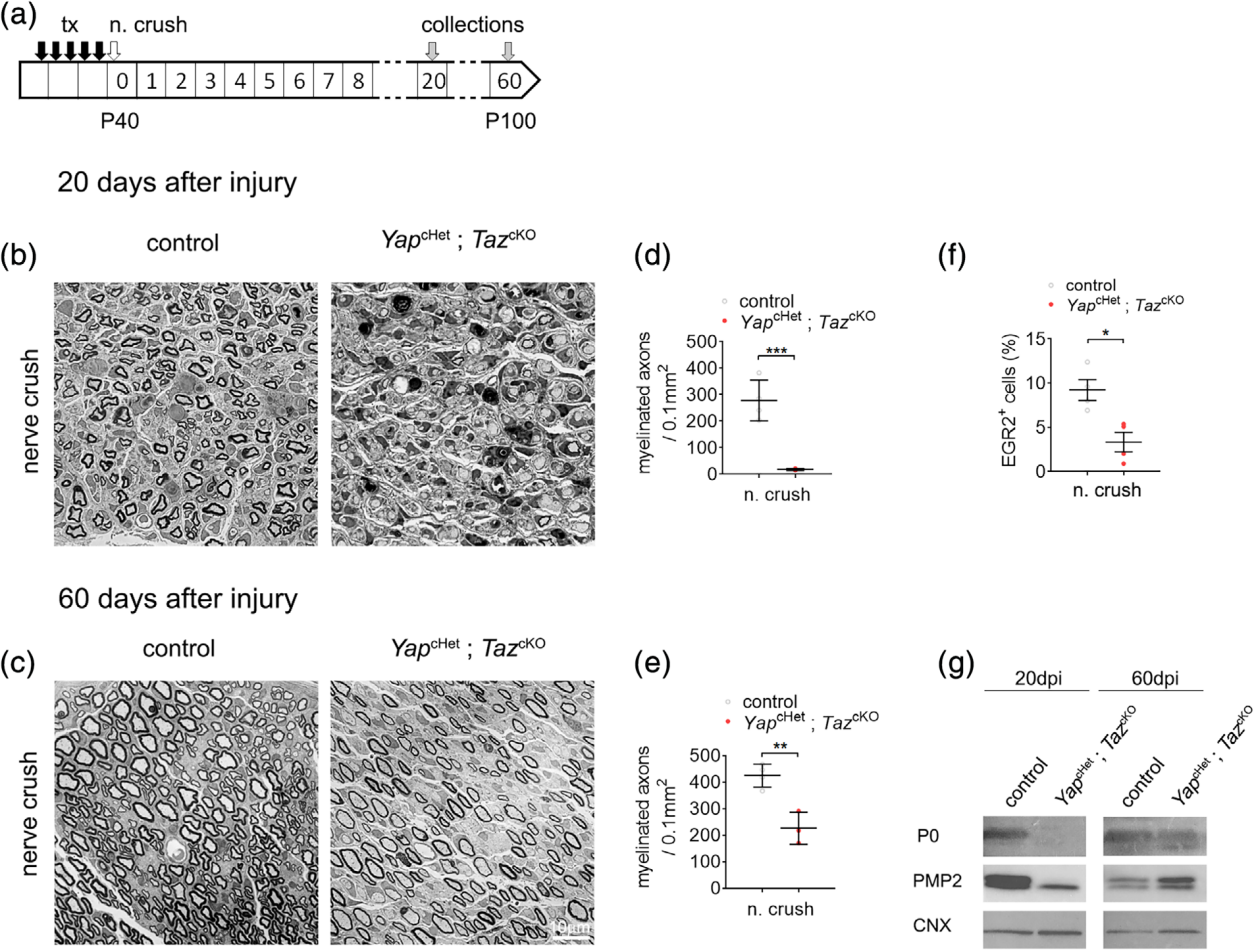
Loss of YAP/TAZ causes severe regeneration defect after injury. (a) Schematic showing experimental procedures analyzing control and *Yap* ^cHet^; *Taz* ^cKO^ sciatic nerves, 20 and 60 days after sciatic nerve crush injury (n. crush). Tamoxifen (tx) was injected before nerve crush injury. (b,c) Semithin sections of the distal sciatic nerve at 20 (b) and 60 days (c) after sciatic nerve crush injury. At 20 days after injury, *Yap* ^cHet^; *Taz* ^cKO^ sciatic nerves show a severe delay in remyelination, with most axons being unmyelinated axons. At 60 days after injury, myelinated axons can be observed in *Yap* ^cHet^; *Taz* ^cKO^ sciatic nerves. Bars, 10 mm. (d,e) Counts of numbers of myelinated fibers in sciatic nerves at 20 and 60 days after injury show significantly fewer myelinated fibers in *Yap* ^cHet^; *Taz* ^cKO^ sciatic nerves at both time points. (f) Quantification of EGR2‐positive cells in control and *Yap* ^cHet^; *Taz* ^cKO^ sciatic nerves after nerve crush injury. Immunohistochemistry was performed at 20 days after injury. *n* ≥ 3 mice for each genotype and time point. Data are presented as means ± *SEM*. Two‐sided Student's *t* test: ***, *p* ≤ .001; **, *p* ≤ .01; *, *p* ≤ .05. (g) Western blot of P0 and PMP2 in injured sciatic nerves at 20 days and 60 days. Myelin protein (P0 and PMP2) are lower at both 20 days postinjury in *Yap* ^cHet^; *Taz* ^cKO^ compared to control, indicating reduction of myelin production. Calnexin (CNX) is used a loading control [Color figure can be viewed at wileyonlinelibrary.com]

To establish the role of YAP and TAZ in SCs following sciatic nerve injury, *Yap* ^cHet^; *Taz* ^cKO^ animals were injected with tamoxifen at postnatal day 40 and underwent unilateral sciatic nerve crush as described in previous report (Savastano et al., 2014). Myelin thickness, the number of myelinated axons, expression level of myelin protein (P0, PMP2) were assessed at 20‐ and 60‐days postinjury (20 and 60 dpi) (Figure 2). At 20 dpi, semithin transverse section results demonstrate a severe nerve regeneration defect in *Yap* ^cHet^; *Taz* ^cKO^ crushed sciatic nerves with nearly no myelinated axons observed (Figure 2b). Western blotting for P0 and PMP2 in *Yap* ^cHet^; *Taz* ^cKO^ crushed sciatic nerves at 20 dpi showed that the severe defect in remyelination was associated with the absence of P0 and decrease of PMP2 protein level at 20 dpi, in contrast to their high expression in control crushed nerves (Figure 2g). EGR2‐positive cells were also significantly lower in *Yap* ^cHet^; *Taz* ^cKO^ crushed nerves 20 dpi compare to control crushed nerves (Figure 2f). At 60 dpi, *Yap* ^cHet^; *Taz* ^cKO^ crushed nerves still present an incomplete remyelination process, with about half of axons remyelinated. The ongoing remyelination was associated with higher levels of myelin protein P0 and PMP2 when compared to 20 dpi (Figure 2g). These results confirm the important role of YAP and TAZ during remyelination observed in a recent study using the complete ablation of *Yap* and *Taz* (Grove et al., 2020); validating the use of the compound model *Yap* ^cHet^; *Taz* ^cKO^ to investigate the long‐term function of YAP and TAZ in remyelination. Since one copy of *Yap* is not enough to compensate for the lack of *Taz*, this suggests that similar to its role during SC development (Deng et al., 2017; Grove et al., 2017; Poitelon et al., 2016), *Taz* has a more prominent role in nerve repair compare to *Yap* (Grove et al., 2020). Finally, these data show that loss of YAP and TAZ expression, at least up to 60 dpi, induces a severe remyelination defect but does not constitute a complete block in peripheral nerve regeneration process.

**FIGURE 3:**
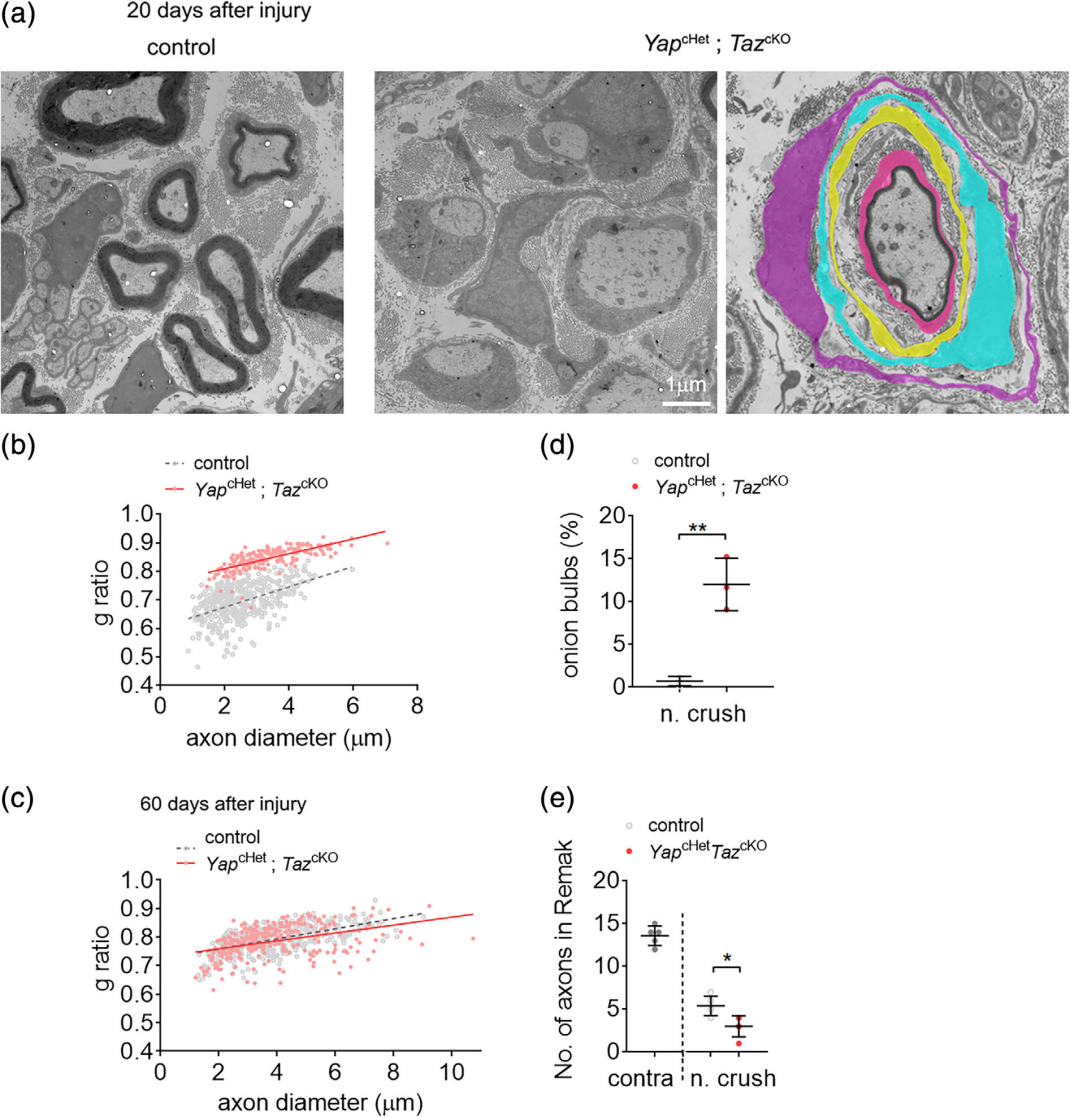
Remyelination and morphological analysis of control and *Yap* ^cHet^; *Taz* ^cKO^ nerves after injury. (a) Transmission electron micrograph pictures of control and *Yap* ^cHet^; *Taz* ^cKO^ sciatic nerves at 20 and 60 days after nerve crush injury. *Yap* ^cHet^; *Taz* ^cKO^ features a panel with unmyelinated axons (left) and an onion bulb (right). Schwann cells in the onion bulb were pseudo‐colored. Bars, 1 μm. (b,c) G ratio versus axon diameter scatter plot graphs for control and *Yap* ^cHet^; *Taz* ^cKO^ sciatic nerves at 20 and 60 days after injury. At 20 days after injury all myelinated axons were counted for *Yap* ^cHet^; *Taz* ^cKO^. (d) Counts of onion bulbs in sciatic nerves of control and *Yap* ^cHet^; *Taz* ^cKO^ at 20 days after injury. No onions bulbs were observed in uninjured nerves or at 60 days after injury. Numerous onion bulbs were observed in *Yap* ^cHet^; *Taz* ^cKO^. *n* = 3 mice. (e) Counts of axons in Remak bundles in sciatic nerves of control and *Yap* ^cHet^; *Taz* ^cKO^ at 60 days after injury and contralateral nerve. Fewer axons in Remak bundles were counted in *Yap* ^cHet^; *Taz* ^cKO^ sciatic nerves. *n* ≥ 3 mice for each genotype and time point. Data are presented as means ± *SEM*. Two‐sided Student's *t* test: **, *p* ≤ .01; *, *p* ≤ .05. No statistical analysis was performed on (b) [Color figure can be viewed at wileyonlinelibrary.com]

We also assessed ultrastructure analysis using electron microscopy in *Yap* ^cHet^; *Taz* ^cKO^ and control crushed nerves. At 20 dpi, by contrast to control nerves, a significant number of SCs are arrested in promyelinating phase (Figure 3a, middle panel) or form onion bulb‐like structures (Figure 3a, right panel) with multiple SCs forming concentric processes around a central SC‐axon unit (Dyck, 1969; Pleasure & Towfighi, 1972; Pollard, King, & Thomas, 1975) (Figure 3a,c). At 60 dpi, *Yap* ^cHet^; *Taz* ^cKO^ nerves are no longer presenting onion bulbs, however there are significantly lower number of axons in Remak bundles compare to control crushed nerves (Figure 3e). Surprisingly, at 60 dpi myelin thickness in *Yap* ^cHet^; *Taz* ^cKO^ (g ratio 0.785 ± 0.01) is comparable to the myelin thickness seen in control crushed nerves (g ratio 0.788 ± 0.009) (Figure 3c). These data suggest that once repair SCs are able to differentiate into myelinating SCs, myelinated fibers get the proper amount of myelin.

**FIGURE 4:**
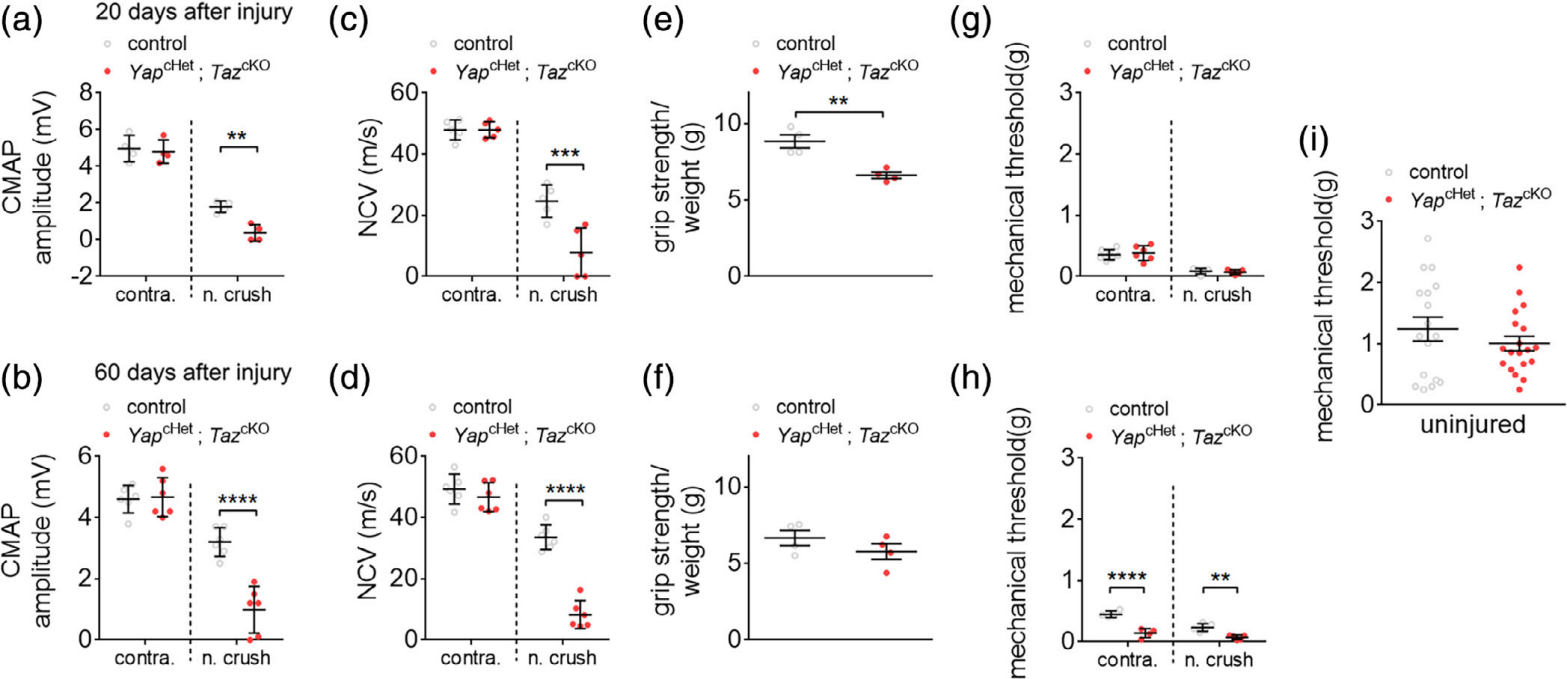
YAP/TAZ‐null animal display diminished functional recovery. (a,b) Measurements of compound muscle action potential (CMAP) of control and *Yap* ^cHet^; *Taz* ^cKO^ animals from contralateral control and side of nerve crush at 20 and 60 days after nerve crush injury. (c,d) Measurements of nerve conduction velocity (NCV) of control and *Yap* ^cHet^; *Taz* ^cKO^ animals from contralateral control and side of nerve crush at 20 and 60 days after nerve injury. (e,f) Measurements of grip strength of control and *Yap* ^cHet^; *Taz* ^cKO^ animals from contralateral control and side of nerve crush at 20 and 60 days after nerve injury. Grip strength measurement were normalized to the weight of the mice. (g–i) Mechanical threshold measurements of control and *Yap* ^cHet^; *Taz* ^cKO^ animals from contralateral and nerve crush side at 20 and 60 days after nerve injury (g,h). Mechanical threshold in uninjured control and *Yap* ^cHet^; *Taz* ^cKO^ animals at 60 days of age (i). *n* ≥ 4 mice for each genotype and time point. Data are presented as means ± *SEM*. Two‐sided Student's *t* test: ****, *p* ≤ 0.0001; ***, *p* ≤ 0.001; **, *p* ≤ .01 [Color figure can be viewed at wileyonlinelibrary.com]

To evaluate how the loss of YAP and TAZ affects the nerve function during remyelination, the CMAP amplitude and nerve conduction velocity were measured in *Yap* ^cHet^; *Taz* ^cKO^ and control crushed nerves at 20 and 60 dpi (Figure 4a–d). As another evaluation for nerve functional regeneration, we utilized behavior tests to evaluate grip strength (grip test) and mechanical allodynia (Von Frey test) in *Yap* ^cHet^; *Taz* ^cKO^ and control mice hindlimbs at 20 and 60 dpi (Figure 4e–i). Nerve conduction strength and speed are both reduced in *Yap* ^cHet^; *Taz* ^cKO^ mice at 20 and 60 dpi (Figure 4a–d). The *Yap* ^cHet^; *Taz* ^cKO^ mice exhibit lower grip strength at 20 dpi, but also lower mechanical allodynia at 60 dpi compare to control mice (Figure 4e–h). Overall, these results show that nerve injury‐derived defects in nerve conduction, grip strength, and mechanical allodynia significantly worsened due to the loss of *Yap* and *Taz* expression in SCs.

### 3.3 The failure of *Yap*^cHet^; *Taz*^cKO^ SCs to remyelinate is associated with c‐JUN dysregulation

**FIGURE 5:**
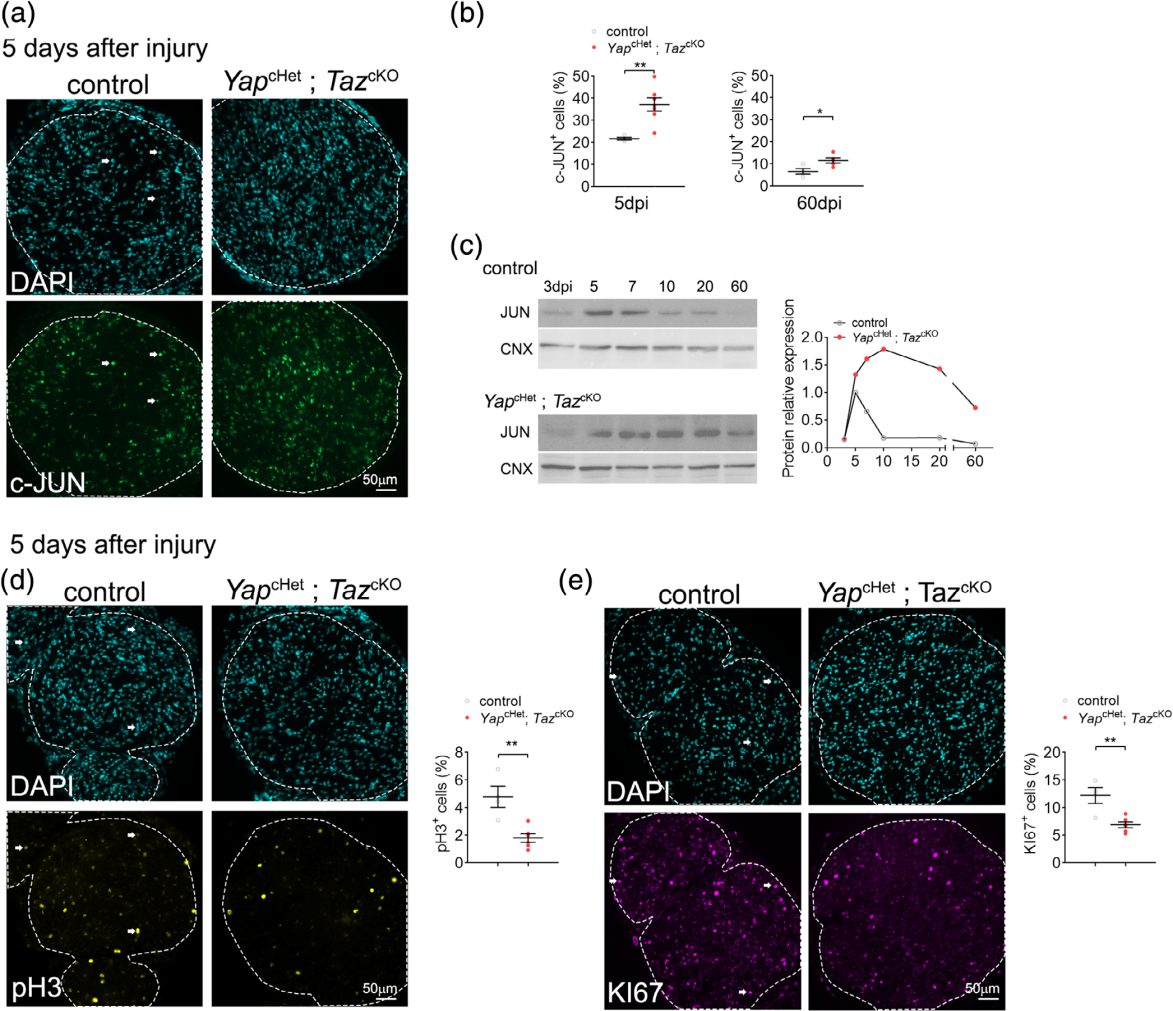
Repair Schwann cell (SC) ablated for YAP/TAZ show decreased proliferation and hyperactivation of c‐JUN. (a–c) Labeling and quantification of JUN‐positive cells in control and *Yap* ^cHet^; *Taz* ^cKO^ sciatic nerves after nerve crush injury. Immunohistochemistry was performed at 5 and 60 days after injury (a‐b) and Western were performed from 3 to 60 days after injury (c). JUN levels are higher in *Yap* ^cHet^; *Taz* ^cKO^ sciatic nerves as early as 5 days after injury and remain higher up to 60 days after injury. Calnexin (CNX) is used a loading control. (d) Labeling and quantification of pH3‐positive cells in control and *Yap* ^cHet^; *Taz* ^cKO^ sciatic nerves 5 days after nerve crush injury. (e) Labeling and quantification of KI67‐positive cells in control and *Yap* ^cHet^; *Taz* ^cKO^ sciatic nerves 5 days after nerve crush injury. Bars, 50 μm. *n* ≥ 4 mice for each genotype and time point. Data are presented as means ± *SEM*. Two‐sided Student's *t* test: **, *p* ≤ .01 [Color figure can be viewed at wileyonlinelibrary.com]

In response to injury, the Schwan cells differentiate into repair SCs which proliferate and activate an extensive gene reprogramming such as the upregulation of c‐JUN, an important transcription factor of the repair program (Arthur‐Farraj et al., 2012; Fazal et al., 2017; Hantke et al., 2014; Jessen & Mirsky, 2016; Klein, Groh, Wettmarshausen, & Martini, 2014; Painter et al., 2014). We evaluated how the loss of YAP and TAZ affect the SC differentiation and proliferation by quantifying positive cells for c‐JUN and for the marker of proliferation pH 3 and KI67 respectively (Figure 5). Using immunolabeling, the percentage of c‐JUN positive cells measured in the *Yap* ^cHet^; *Taz* ^cKO^ crushed nerve are significantly increased both at 5 and 60 dpi (Figure 5a,b). In addition, using western blot, high expression of c‐JUN in the *Yap* ^cHet^; *Taz* ^cKO^ crushed nerve lysates are observed from 5 to 60 dpi compare to control crushed nerves (Figure 5c). Immunolabeled cells for both pH 3 and KI67 are also significantly decreased in *Yap* ^cHet^; *Taz* ^cKO^ crushed nerve (Figure 5d,e), indicating slower proliferation.

After injury, the repair programing inducing demyelination activates macrophages influx to the nerve to clear out the cellular debris (Jessen & Mirsky, 2016). We evaluated if the loss of YAP and TAZ affect macrophages influx in the nerve by quantifying positive cells for F4/80 a marker of macrophages. The percentage of F4/80 positive cells measured by immunolabeling in the *Yap* ^cHet^; *Taz* ^cKO^ crushed nerve is not different to the control crushed nerves both at 5 and 60 dpi (Figure S3a,b). To evaluate if the poor remyelination seen after the loss of YAP and TAZ was also due to a defect in axonal regrowth, we quantified GAP43 a marker of axonal regeneration (Ma et al., 2011). GAP43 quantifications by Western blotting in the *Yap* ^cHet^; *Taz* ^cKO^ crushed nerve lysates from 5 to 60 dpi compare to control crushed nerves indicate no difference (Figure S3c). Overall, these results indicate that the lack of remyelination seen in *Yap* ^cHet^; *Taz* ^cKO^ crushed nerve is associated to the maintained expression of c‐JUN in SCs and a lower proliferation rate of repair SCs compare to the control nerves.

### 3.4 Repair program efficiency relies on YAP and TAZ expression during SC differentiation

**FIGURE 6:**
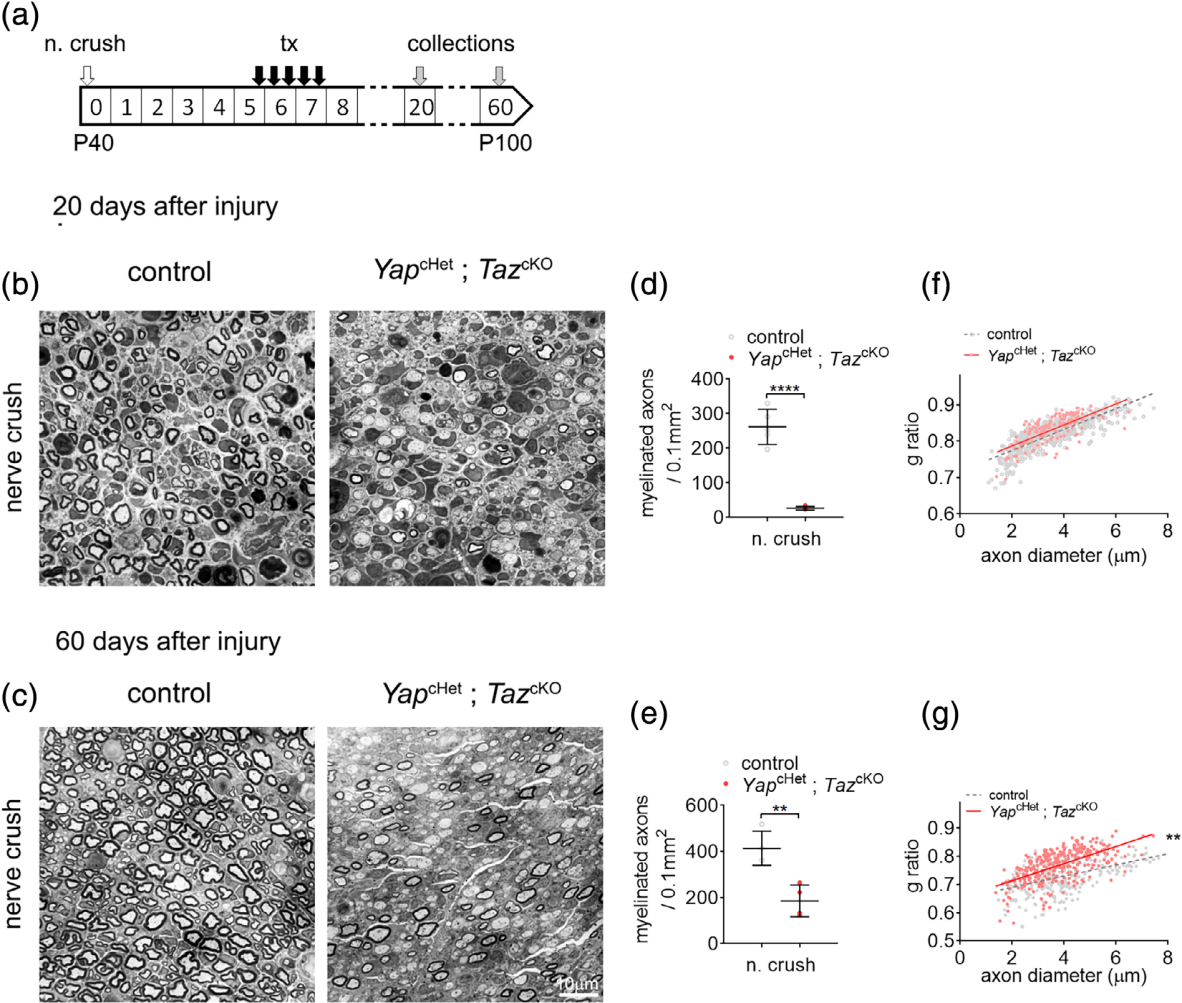
YAP/TAZ are required for remyelination. (a) Schematic showing experimental procedures analyzing control and *Yap* ^cHet^; *Taz* ^cKO^ sciatic nerves, 20 and 60 days after sciatic nerve crush injury (n. crush). Tamoxifen (tx) was injected 5 days after nerve crush injury. (b–e) Semithin sections of the distal sciatic nerve at 20 (b) and 60 days (c) after sciatic nerve crush injury. At 20 and 60 days after injury, *Yap* ^cHet^; *Taz* ^cKO^ sciatic nerves show a severe delay in remyelination, with most axons being unmyelinated axons (d,e). (f,g) G ratio versus axon diameter scatter plot graphs for control and *Yap* ^cHet^; *Taz* ^cKO^ sciatic nerves at 20 and 60 days after injury. At 20 days after injury all myelinated axons were counted for *Yap* ^cHet^; *Taz* ^cKO^. Bars, 10 mm. *n* ≥ 3 mice for each genotype and time point. Data are presented as means ± *SEM*. Two‐sided Student's *t* test: ****, *p* ≤ 0.0001; **, *p* ≤ .01. No statistical analysis was performed on (f) [Color figure can be viewed at wileyonlinelibrary.com]

To assess to role of YAP and TAZ in SCs during remyelination, we ablated *Yap* and *Taz* after differentiation into repair SCs (Arthur‐Farraj et al., 2017). P40 *Yap* ^cHet^; *Taz* ^cKO^ animals were injected 5 to 7 days after unilateral nerve crush (Figure 6a). This approach allows us to better characterize if YAP and TAZ are required during SC differentiation into repair SCs or during repair SC redifferentiation into myelinating SCs. Myelin thickness and the number of myelinated axons were assessed at 20 and 60 dpi (Figure 6b–g). At 20 dpi, semithin transverse sections have a severe nerve regeneration defect in *Yap* ^cHet^; *Taz* ^cKO^ crushed sciatic nerves with very few remyelinated axons (Figure 6b–f). Interestingly, onion bulbs, a hallmark observed in our previous paradigm (YAP and TAZ ablated prior injury) (Figure 3d), are not observed when tamoxifen is injected 5–7 days after nerve crush. At 60 dpi, *Yap* ^cHet^; *Taz* ^cKO^ crushed nerves present an incomplete remyelination process, with about half remyelinated axons and improper myelin thickness (g ratio 0.766 ± 0.003) in comparison myelin thickness seen in control crushed nerves (g ratio 0.728 ± 0.006) (Figure 6c–g). We also demonstrated that ablating *Yap* and *Taz* before and after injury show similar remyelination defects; were nearly no remyelinated fibers are observed. Taken together, these present data indicate that myelination defects observed in *Yap* ^cHet^; *Taz* ^cKO^ are likely to be caused by a defect in the transition from repair SCs into myelinating SCs.

## 4 DISCUSSION

The ablation *Yap* and *Taz* in mouse SCs prevents the normal development of peripheral nerves due to the transcriptional dysregulation of genes regulating axonal sorting, SC proliferation and myelination (Deng et al., 2017; Grove et al., 2017; Poitelon et al., 2016). However, the role of YAP and TAZ in myelin maintenance is unclear due to conflicting reports, describing YAP and TAZ as being either essential or non‐essential for myelin maintenance when ablated in adult nerves (Deng et al., 2017; Grove et al., 2017). Further, the role of YAP and TAZ in myelin maintenance was only limited to short‐term studies, given the short lifespan (2–3 weeks) of the *Yap* and *Taz* double knockout mice (Deng et al., 2017; Grove et al., 2017). In the present study, we used *Yap* ^cHet^; *Taz* ^cKO^ mouse model which is a compound ablation model of *Yap* and *Taz* with a normal lifespan. Our results corroborated the observations of Deng et al., and show no defect in myelin maintenance and no behavioral or functional defects in the *Yap* ^cHet^; *Taz* ^cKO^ mice up to 60 days after tamoxifen injections.

*Yap* ^cHet^; *Taz* ^cKO^ animals showed no preexisting phenotype which prompted us to use this model to study the role of YAP and TAZ in peripheral nerve regeneration and remyelination. After nerve crush, SCs distal to the crush site lose contact with the axons as they degenerate. Mature SCs can adopt a transient repair phenotype converting Remak SC and myelinating SCs into repair SCs that promote a suitable environment for nerve regeneration. The transition is promoted by the (a) downregulation of genes regulating myelination such as Egr2 and (b) upregulation of c‐JUN, a master regulator for the trans‐differentiation into repair SCs (Arthur‐Farraj et al., 2012; Fontana et al., 2012; Parkinson et al., 2008; Pereira, Lebrun‐Julien, & Suter, 2012). Repair SCs are notably involved in myelin autophagy, the expression of cytokines that recruit macrophages for additional myelin clearance, and the expression of trophic factors to stimulate axonal regrowth. During axonal regrowth, repair SCs redifferentiate into Remak SCs and myelinating SCs to enwrap newly grown axons (Arthur‐Farraj et al., 2012; Jessen & Mirsky, 2019). SCs in *Yap* ^cHet^; *Taz* ^cKO^ are able to successfully initiate the transcriptional repair program through increase in c‐JUN expression and decrease of EGR2‐postive SCs. However, we observed an almost complete failure of SCs to remyelinate axons in the compound *Yap* ^cHet^; *Taz* ^cKO^ mice at 20 dpi. These results are in accordance with a recent report using inducible *Yap* and *Taz* double knockout mice and investigating peripheral nerve regeneration up to 2 weeks after nerve injury (Grove et al., 2020). We further observed at 60 dpi a significant increase of myelin protein amount and a higher number of remyelinated fibers, indicating that nerve regeneration and remyelination in *Yap* ^cHet^; *Taz* ^cKO^ are not completely impaired and that over time YAP and TAZ functional defect in SCs is compensated. Compensation by the remaining allele *Yap* is possible, but it remains unlikely based on the predominant role of TAZ compare to YAP during development (Deng et al., 2017; Grove et al., 2017; Poitelon et al., 2016) and on previous report showing nearly no upregulation of YAP expression in nerves after crush (Grove et al., 2020; Mindos et al., 2017).

Our analyses also showed that SCs ablated for YAP and TAZ were able to redifferentiate into functional repair SCs. We did not observe any defects in myelin clearance, recruitment of F4/80‐positive cells nor GAP43 protein level in the *Yap* ^cHet^; *Taz* ^cKO^ crushed nerves compare to control crushed nerves, suggesting that the stark remyelination defects observed in *Yap* ^cHet^; *Taz* ^cKO^ are not caused by non‐cell‐autonomous defects in macrophages or neurons (Mindos et al., 2017). However, we observed a decrease in SC proliferation and a sustained expression of c‐JUN from 5 dpi up to 60 dpi in *Yap* ^cHet^; *Taz* ^cKO^ crushed nerve, while c‐JUN expression is known to peak around 5 dpi after crush injury in control nerves and decrease dramatically after (Painter et al., 2014). These results challenge recent data from Grove et al. indicating that both proliferation and c‐JUN protein level are not affected in crushed nerves of *Yap* and *Taz* double knockouts. Since both studies used *Yap* f/f and *Taz* f/f animals from different sources, it is possible difference in the design of the *Yap* and/or *Taz* floxed animals account for the variability of phenotype (Reginensi et al., 2013; Xin et al., 2013). It is also possible that haploinsufficiency of *Yap* in *Yap* ^cHet^; *Taz* ^cKO^ has a dominant‐negative effect on SC transcriptional regulation of c‐JUN and proliferation regulators.

*Yap* and *Taz* are known in many cell types, including SCs, to promote transcriptional programs for proliferation by upregulating genes involved in cell cycle control (Deng et al., 2017; Grove et al., 2017; Ma, Meng, Chen, & Guan, 2019; Poitelon et al., 2016). Similarly, downregulation of EGR2 is necessary for the differentiation of mature SCs into repair SCs (Warner et al., 1998), which in turn has been shown to inhibit c‐JUN expression (Parkinson et al., 2004; Svaren & Meijer, 2008). *Yap* and *Taz* are known to positively regulate EGR2 in SCs (Deng et al., 2017; Grove et al., 2017;Lopez‐Anido et al., 2016; Poitelon et al., 2016). Our data show at 20 dpi that EGR2‐positive cells are lower in *Yap* ^cHet^; *Taz* ^cKO^ crushed nerves compared to control crushed nerves. Since it is known that c‐JUN and EGR2 repress each other (Parkinson et al., 2008), it is possible that the c‐JUN level increase in *Yap* ^cHet^; *Taz* ^cKO^ crushed nerves is due to reduced inhibition by EGR2. Overall, our data are in accordance with c‐JUN and EGR2 cross‐antagonistic functional relationship (Parkinson et al., 2008). However, the effect of elevated level of c‐JUN has been shown to be dosage dependent. During nerve regeneration, while the mild overexpression of c‐JUN (c‐Jun OE/+) accelerates SC‐mediated myelin breakdown, c‐JUN elevated expression causes a significant delay in remyelination and a decrease in myelin thickness (Fazal et al., 2017). Since our observations are similar to c‐JUN overexpressor mice, the dysregulation of c‐JUN level could explain the myelination defect associated to *Yap* ^cHet^; *Taz* ^cKO^ crushed nerves. However, considering that we observe a modest increase level of c‐JUN up to twofold, it is likely that increased c‐JUN expression is not entirely contributing to the defects observed in *Yap* ^cHet^; *Taz* ^cKO^ crushed nerves and that other important YAP/TAZ‐dependent remyelination regulators are being dysregulated, in particular EGR2 (Atanasoski et al., 2006; Kim et al., 2000; Yang et al., 2008). This hypothesis was confirmed by our demonstration that remyelination in *Yap* ^cHet^; *Taz* ^cKO^ crushed nerves is impaired even if *Yap* and *Taz* are ablated after the peak of c‐JUN expression and the transdifferentiation into repair SC.

An interesting point we observed is the elevated formation of onion bulbs in the *Yap* ^cHet^; *Taz* ^cKO^ crushed nerves. Onion bulb formation is the pathological hallmark of inherited peripheral neuropathies such as in Charcot–Marie–Tooth Type 1A (Dyck, 1966; Fledrich et al., 2019). During Wallerian degeneration, SCs lose contact with degenerating axons and initiate an autocrine glial NRG1‐I‐mediated survival (Stassart et al., 2013). A recent report associated the increase formation of onion bulbs in chronic neuropathies to an axonal‐SC interaction impairment (Fledrich et al., 2019). It was proposed that the transient autocrine SC NRG1‐I‐mediated survival, known to be beneficial during acute nerve injury, is sustained in chronic demyelinating neuropathies which leads to the increase in onion bulb formation (Fledrich et al., 2019). The onion bulb formation was correlated to the autocrine glial NRG1‐I expression level and to an increase expression of c‐JUN level in myelinating and supernumerary SCs in neuropathic models (Fledrich et al., 2019). Remarkably, in our study YAP and TAZ ablation known to impair axon/SC interaction during development (Lopez‐Anido et al., 2016; Poitelon et al., 2016), dysregulates c‐JUN expression in dedifferentiated SCs after injury. Therefore it is possible that after injury the increase formation of onion bulbs in *Yap* ^cHet^; *Taz* ^cKO^ crushed nerves is the consequence of a sustained expression of c‐JUN in dedifferentiated SC ablated for YAP and TAZ (Fazal et al., 2017; Fledrich et al., 2019).

In conclusion, while YAP and TAZ are major regulators of nerve development, using a compound *Yap* ^cHet^; *Taz* ^cKO^ model we have demonstrated that YAP and TAZ are not necessary for short and long‐term myelin maintenance. Our findings also reveal that in absence of YAP and TAZ, mature SCs are successfully reprogrammed into repair SCs after nerve injury and ensure the survival and regrowth of injured neurons overtime. We have shown that repair SCs lacking YAP and TAZ have reduced proliferation, sustained c‐JUN expression and lose the ability to redifferentiate into myelinating SCs. Together our results suggest that YAP and TAZ are potential targets for SC myelination and remyelination programming and further studies should target the Hippo pathway in inherited demyelinating disorders.

## CONFLICT OF INTEREST

The authors declare no conflict of interests.

## AUTHOR CONTRIBUTIONS

**Sophie Belin** and **Yannick Poitelon**: Designed research, analyzed data, and wrote the manuscript. **Haley Jeanette**, **Sophie Belin**, and **Yannick Poitelon**: Performed experiments with **Jacob Herron**, **Abigail Hellman** and **Urja Bhatia** assistance. **Marziali N. Leandro**: Performed electron microscopy analyses. **Maria Laura Feltri**: Contributed to generation of mutant mice at the beginning of the project and critically reviewed the manuscript. **Ashley M. Kopec**: Critically reviewed the manuscript.

## Supporting information


**Figure S1** Myelin maintenance is not affected by compound ablation of YAP and TAZ. (**a**) Schematic showing experimental procedures analyzing intact nerves of *Yap* ^f/+^; *Taz* ^f/f^; *Sox10*‐Cre^ER^ sciatic nerves 60 days after oil (−tx) or tamoxifen (+tx) injections. (**b**) Semithin sections of *Yap* ^f/+^; *Taz* ^f/f^; *Sox10*‐Cre^ER^ sciatic nerves 60 days after oil (−tx) or tamoxifen (+tx) injections. Bars, 10 μm. (**c**) Counts of numbers of myelinated fibers in *Yap* ^f/+^; *Taz* ^f/f^; *Sox10*‐Cre^ER^ sciatic nerves 60 days after oil (−tx) or tamoxifen (+tx) injections. (**d**) G ratio versus axon diameter scatter plot graphs from myelinated fibers in *Yap* ^f/+^; *Taz* ^f/f^; *Sox10*‐Cre^ER^ sciatic nerves 60 days after oil (−tx) or tamoxifen (+tx) injections. n ≥ 3 mice for each genotype and time point. Data are presented as means ± SEM.
**Figure S2**: Double ablation of YAP/TAZ in neural crest derivatives lead to animal death. (**a**) Schematic showing experimental procedures analyzing *Yap* ^cKO^; *Taz* ^cKO^ sciatic nerves, 13 days after oil or tamoxifen injections. (**b**) Semithin sections of *Yap* ^cKO^; *Taz* ^cKO^ sciatic nerves 13 days after oil or tamoxifen injections. Bars, 10 μm. (**c**) Body weight from *Yap* ^cKO^; *Taz* ^cKO^ animals after oil or tamoxifen injections and wild‐type animal after tamoxifen injections. n ≥ 6 mice for each genotype and time point. Data are presented as means ± SEM. Two‐sided Student's *t* test: ****, *p* ≤ 0.0001; ***, *p* ≤ 0.001.
**Figure S3**: Loss of YAP/TAZ does not affect the axonal regrowth or macrophages infiltration. (**a**) Labeling and quantification of F4/80‐positive macrophages in control and *Yap* ^cHet^; *Taz* ^cKO^ sciatic nerves at 5 and 60 days after nerve crush injury. n ≥ 4 mice for each genotype and time point. Bars, 50 μm. Data are presented as means ± SEM. (**b**) Western blots of distal control and *Yap* ^cHet^; *Taz* ^cKO^ nerves at 3, 5, 7, 10, 20 and 60 days after nerve injury. Values represent normalized expression against the control for each time point. Calnexin (CNX) is used a loading control.

## Data Availability

The data that support the findings of this study are available from the corresponding author upon reasonable request.
