# Supplementary material for: YAP and TAZ regulate Schwann cell proliferation and differentiation during peripheral nerve regeneration
Source: Glia. 2020 Dec 18;69(4):1061–74. doi: 10.1002/glia.23949 (PMC7898398; doi:10.1002/glia.23949)
Supplement: Supplementary file 1 — Figure S1 Myelin maintenance is not affected by compound ablation of YAP and TAZ. (a) Schematic showing experimental procedures analyzing intact nerves of Yap f/+; Taz f/f; Sox10‐CreER sciatic nerves 60 days after oil (−tx) or tamoxifen (+tx) injections. (b) Semithin sections of Yap f/+; Taz f/f; Sox10‐CreER sciatic nerves 60 days after oil (−tx) or tamoxifen (+tx) injections. Bars, 10 μm. (c) Counts of numbers of myelinated fibers in Yap f/+; Taz f/f; Sox10‐CreER sciatic nerves 60 days after oil (−tx) or tamoxifen (+tx) injections. (d) G ratio versus axon diameter scatter plot graphs from myelinated fibers in Yap f/+; Taz f/f; Sox10‐CreER sciatic nerves 60 days after oil (−tx) or tamoxifen (+tx) injections. n ≥ 3 mice for each genotype and time point. Data are presented as means ± SEM. Figure S2: Double ablation of YAP/TAZ in neural crest derivatives lead to animal death. (a) Schematic showing experimental procedures analyzing Yap cKO; Taz cKO sciatic nerves, 13 days after oil or tamoxifen injections. (b) Semithin sections of Yap cKO; Taz cKO sciatic nerves 13 days after oil or tamoxifen injections. Bars, 10 μm. (c) Body weight from Yap cKO; Taz cKO animals after oil or tamoxifen injections and wild‐type animal after tamoxifen injections. n ≥ 6 mice for each genotype and time point. Data are presented as means ± SEM. Two‐sided Student's t test: ****, p ≤ 0.0001; ***, p ≤ 0.001. Figure S3: Loss of YAP/TAZ does not affect the axonal regrowth or macrophages infiltration. (a) Labeling and quantification of F4/80‐positive macrophages in control and Yap cHet; Taz cKO sciatic nerves at 5 and 60 days after nerve crush injury. n ≥ 4 mice for each genotype and time point. Bars, 50 μm. Data are presented as means ± SEM. (b) Western blots of distal control and Yap cHet; Taz cKO nerves at 3, 5, 7, 10, 20 and 60 days after nerve injury. Values represent normalized expression against the control for each time point. Calnexin (CNX) is used a loading control. [file GLIA-69-1061-s001.docx]

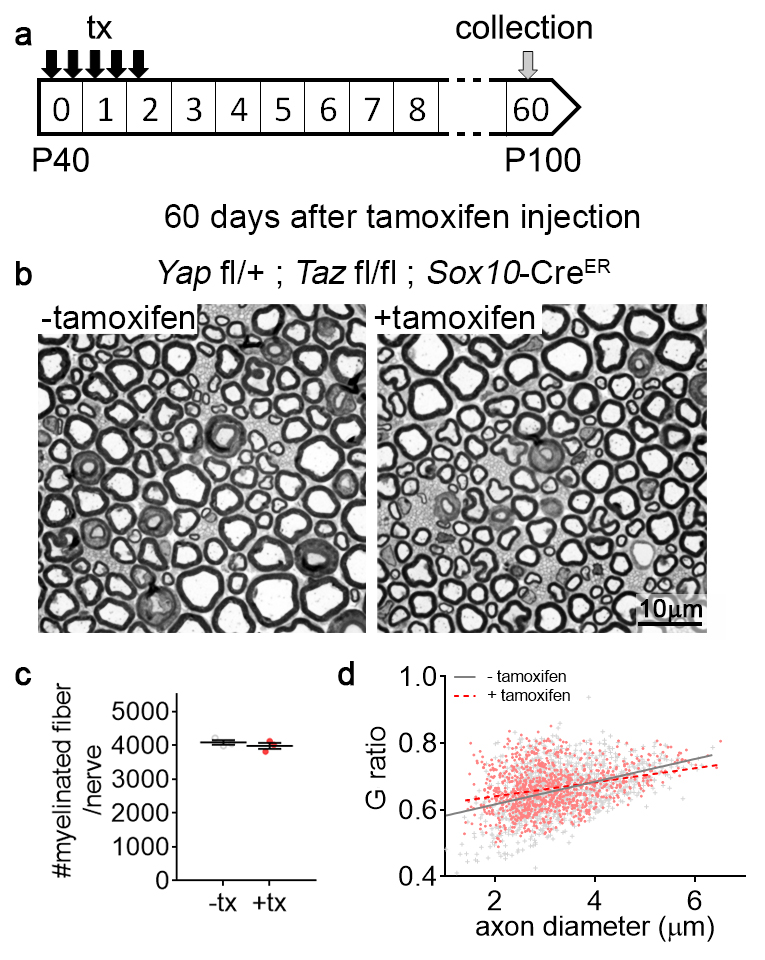


**Fig.S1**: Myelin maintenance is not affected by compound ablation of YAP and TAZ. (**a**) Schematic showing experimental procedures analyzing intact nerves of *Yap*^f/+^ ; *Taz*^f/f^ ; *Sox10*-Cre^ER^ sciatic nerves 60 days after oil (-tx) or tamoxifen (+tx) injections. (**b**) Semithin sections of *Yap*^f/+^ ; *Taz*^f/f^ ; *Sox10*-Cre^ER^ sciatic nerves 60 days after oil (-tx) or tamoxifen (+tx) injections. Bars, 10 µm. (**c**) Counts of numbers of myelinated fibers in *Yap*^f/+^ ; *Taz*^f/f^ ; *Sox10*-Cre^ER^ sciatic nerves 60 days after oil (-tx) or tamoxifen (+tx) injections. (**d**) G ratio versus axon diameter scatter plot graphs from myelinated fibers in *Yap*^f/+^ ; *Taz*^f/f^ ; *Sox10*-Cre^ER^ sciatic nerves 60 days after oil (-tx) or tamoxifen (+tx) injections. n ≥ 3 mice for each genotype and time point. Data are presented as means ± SEM.


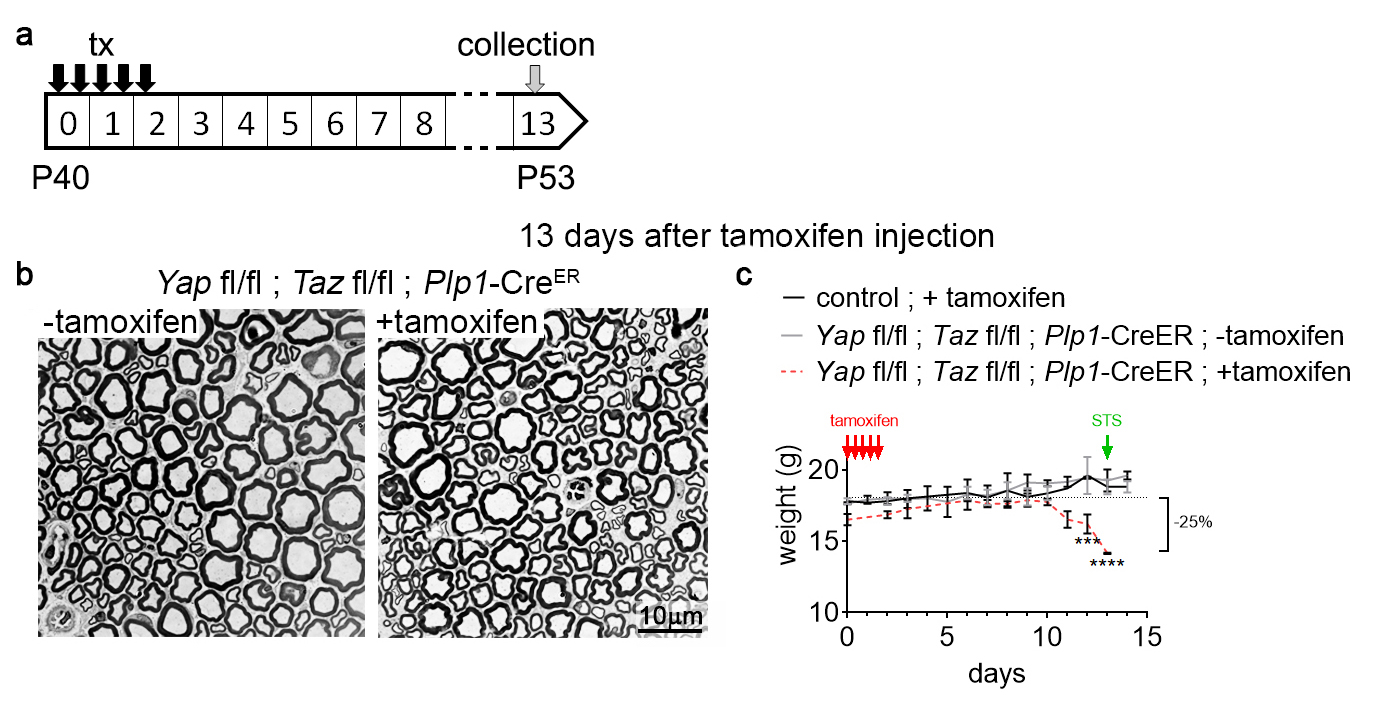


**Fig.S2**: Double ablation of YAP/TAZ in neural crest derivatives lead to animal death. (**a**) Schematic showing experimental procedures analyzing *Yap*^cKO^ ; *Taz*^cKO^ sciatic nerves, 13 days after oil or tamoxifen injections. (**b**) Semithin sections of *Yap*^cKO^ ; *Taz*^cKO^ sciatic nerves 13 days after oil or tamoxifen injections. Bars, 10 µm. (**c**) Body weight from *Yap*^cKO^ ; *Taz*^cKO^ animals after oil or tamoxifen injections and wild-type animal after tamoxifen injections. n ≥ 6 mice for each genotype and time point. Data are presented as means ± SEM. Two-sided Student’s t test: ****, *P* ≤ 0.0001; ***, *P* ≤ 0.001.


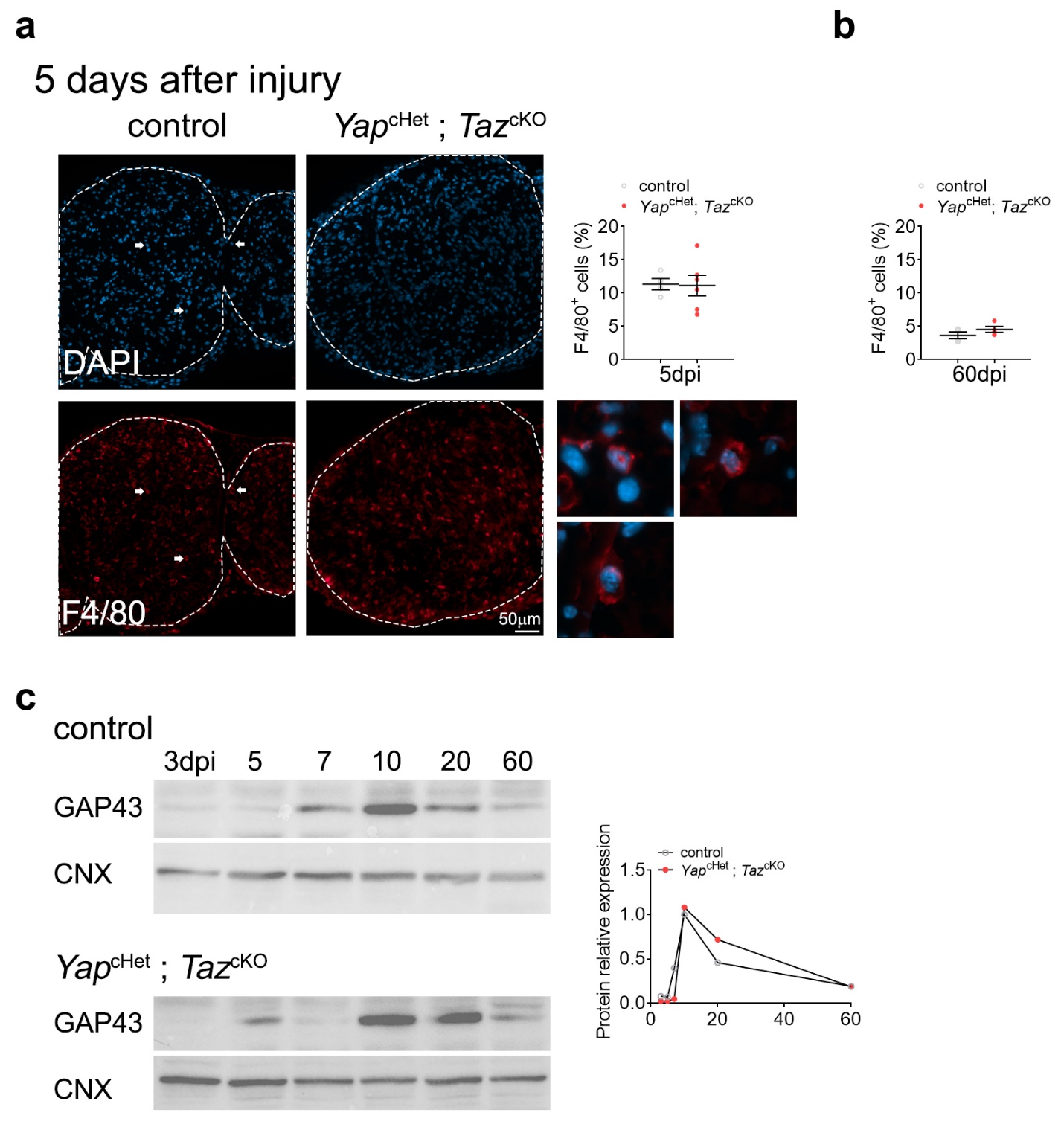


**Fig.S3**: Loss of YAP/TAZ does not affect the axonal regrowth or macrophages infiltration. (**a**) Labeling and quantification of F4/80-positive macrophages in control and *Yap*^cHet^ ; *Taz*^cKO^ sciatic nerves at 5 and 60 days after nerve crush injury. n ≥ 4 mice for each genotype and time point. Bars, 50 μm. Data are presented as means ± SEM. (**b**) Western blots of distal control and *Yap*^cHet^ ; *Taz*^cKO^ nerves at 3, 5, 7, 10, 20 and 60 days after nerve injury. Values represent normalized expression against the control for each time point. Calnexin (CNX) is used a loading control.
